# Anisocytosis is Associated With Short-Term Mortality in COVID-19 and May Reflect Proinflammatory Signature in Uninfected Ambulatory Adults

**DOI:** 10.20411/pai.v5i1.391

**Published:** 2020-10-02

**Authors:** Andrew Hornick, Nour Tashtish, Michael Osnard, Binita Shah, Allison Bradigan, Zainab Albar, Jeffrey Tomalka, Jarrod Dalton, Ashish Sharma, Rafick P. Sekaly, Rana Hejal, Daniel I. Simon, David A. Zidar, Sadeer G. Al-Kindi, On Behalf Of Inaction Study Group.

**Affiliations:** 1 Harrington Heart & Vascular Institute, University Hospitals Cleveland Medical Center; 2 New York VA Harbor Healthcare System and New York University School of Medicine, New York, NY; 3 Seidman Cancer Center, University Hospitals Cleveland Medical Center, Cleveland, OH; 4 Case Western Reserve University, Cleveland, OH; 5 Department of Quantitative Health Sciences, Lerner Research Institute, Cleveland Clinic, Cleveland, OH; 6 Department of Pulmonary and Critical Care, University Hospitals, Cleveland, OH; 7 Louis Stokes Cleveland VA Medical Center, Cleveland, OH

**Keywords:** Covid-19, Anisocytosis, RDW, Erythrocyte Indices, Prognosis, Critical Illness, Cytokines

## Abstract

**BACKGROUND:**

Red cell distribution width (RDW), a measure of anisocytosis, is observed in chronic inflammation and is a prognostic marker in critically ill patients without COVID-19, but data in COVID-19 are limited.

**METHODS:**

Between March 12 and April 19, 2020, 282 individuals with confirmed COVID-19 and RDW available within 7 days prior to COVID-19 confirmation were evaluated. Individuals were grouped by quartiles of RDW. Association between quartiles of RDW and mortality was assessed using the Kaplan-Meier method and statistical significance was assessed using the log-rank test. The association between RDW and all-cause mortality was further assessed using a Cox proportional hazards model. Plasma cytokine levels in uninfected ambulatory adults without cardiovascular disease (n=38) were measured and bivariate Spearman correlations and principle components analysis were used to identify relationships between cytokine concentrations with RDW.

**RESULTS:**

After adjusting for age, sex, race, cardiovascular disease, and hemoglobin, there was an association between RDW and mortality (Quartile 4 vs Quartile 1: HR 4.04 [1.08-15.07]), with each 1% increment in RDW associated with a 39% increased rate of mortality (HR 1.39 [1.21-1.59]). Remote RDW was also associated with mortality after COVID-19 infection. Among uninfected ambulatory adults without cardiovascular disease, RDW was associated with elevated pro-inflammatory cytokines (TNF-α, IL8, IL6, IL1b), but not regulatory cytokines (TGFb).

**CONCLUSIONS:**

Anisocytosis predicts short-term mortality in COVID-19 patients, often predates viral exposure, and may be related to a pro-inflammatory phenotype. Additional study of whether the RDW can assist in the early identification of pending cytokine storm is warranted.

Chronic anisocytosis is a powerful predictor of short-term mortality after COVID-19 infection

## INTRODUCTION

The coronavirus disease 2019 (COVID-19) pandemic rapidly rose to prominence in the United States during the first months of 2020. Numerous studies have sought to identify both the mechanism of severe disease and prognostic methods to identify patients at risk for progression to severe disease and death. Reported data consistently identify dysregulation of inflammatory and thrombotic pathways in patients with severe COVID-19 [1-4]. Studies of the host response reveal a complex immunopathogenesis with failure of type 1 interferon responses [5], hyper-inflammation [6], T cell exhaustion [7], and ineffective adaptive/antibody responses [8] in those with a progressive course. There is a need to develop biomarkers to identify patients at increased risk for the development of a severe clinical COVID-19 course for whom preventive strategies can be targeted.

Red blood cell distribution width (RDW), a measure of the variability of circulating erythrocyte size, is routinely reported as part of a complete blood count, but is not typically used clinically. Elevated RDW is associated with poor outcomes in a wide variety of disease states, including cardiovascular disease, stroke, and critical illness [9-12]. RDW has also been shown to correlate with measures of inflammation in non-COVID-19 settings, including tumor necrosis factor (TN-F)-α [13] in sepsis, interleukin (IL)-6 [14,15] in heart failure and human immunodeficiency virus infection, and high sensitivity C-Reactive protein (hsCRP) and erythrocyte sedimentation rate (ESR) [16,17] in other populations.

The primary aim of this study was to determine whether RDW, assessed prior to infection, was associated with short-term mortality among patients with confirmed COVID-19 in a single large integrated academic health system. We additionally performed an analysis of an uninfected ambulatory cohort to understand the inflammatory correlates of RDW.

## METHODS

In this retrospective study, we studied consecutive patients who tested positive for COVID-19 using a viral polymerase chain reaction (PCR) test between March 12 and April 19, 2020 at University Hospitals Health System. University Hospitals Health System is comprised of 14 hospitals serving over 1 million patients in northeast Ohio. Inclusion criteria included positive COVID-19 PCR test and available RDW within 7 days prior to COVID-19 PCR test. Patients <18 years were excluded. We extracted demographics, medical history (diabetes, hypertension, lung disease, cardiovascular disease, and cancer) and laboratory markers from within 7 days prior to COVID-19 confirmation (leukocyte count, neutrophil count, monocyte count, lymphocyte count, hemoglobin, mean corpuscular volume [MCV], RDW, and platelet count) from the automatically queried electronic medical records. All labs were performed at local clinical facilities and reported in the system-wide electronic medical record system. The primary outcome was defined as all-cause mortality, which was ascertained through review of medical records and linkage with State of Ohio death index files.

The baseline demographics and characteristics of participants grouped by RDW quartiles were compiled as raw numbers and percentages for categorical variables and means and standard deviation for continuous variables. Statistical significance was examined using chi-square and analysis of variance F-tests (overall comparisons) and by trend tests (Mantel-Haenszel test for categorical variables and Spearman rank test for continuous variables). A Wilcoxon paired test was used to compare remote RDW, defined as a value from >7 days prior to testing positive for COVID-19, and acute RDW, defined as a value from <7 days prior to testing positive for COVID-19. The association between RDW and mortality was further investigated using RDW as a continuous variable and penalized smoothed spline with Cox proportional hazards regression models. Association between quartiles of RDW and mortality was assessed using the Kaplan-Meier method and statistical significance was assessed using the log-rank test. To evaluate the association between RDW quartiles and mortality, we constructed three Cox proportional hazard models with increasing adjustment. There is an unadjusted model, as well as a model (model 1) adjusted for age, sex, and race. The second model (model 2) was additionally adjusted for hemoglobin and preexisting cardiovascular disease. To analyze the predictive power of RDW, we performed time-sensitive receiver operating characteristics at 30 days using “survivalROC” function in RStudio. ROC analysis for 30-day mortality was also performed for age as a comparator. Cut-points were identified with 80% sensitivity and 80% specificity. Optimal cut-point was identified using maximal Youden index (highest sensitivity+specificity).

Given the results of the primary analysis described above, we conducted a post-hoc study of cytokines in ambulatory volunteers without a history of cardiovascular disease to generate hypotheses of the mechanism of action on which RDW is reflective. Inclusion criteria for participation in the study included ≥18 years with no history of atherosclerotic cardiovascular disease. To determine the cytokines that relate to RDW, we measured 23 plasma cytokines in uninfected ambulatory adults (n=38) using electrochemiluminescence (Meso Scale Discovery, Gaithersburg, Maryland, USA). Twenty-two of these cytokines gave non-zero levels and were analyzed using Spearman correlations, hierarchical clustering, and principal component analyses. All analyses were performed in Statistical Package for Social Sciences (SPSS, version 23, IBM, NY) and RStudio version 1.2.1335 (R project, Austria). Informed consent was obtained from all human subject study participants, and University Hospitals' Institutional Review Board reviewed and provided approval for the study protocol.

## RESULTS

A total of 282 patients were included. Median [quartiles] age was 63 [53-75] years; 137 (49%) were male and 173 (61.3%) were white. The majority of patients were hospitalized (n=175, 62%), 74 (26%) were discharged from the emergency department, and the remaining patients (n=33, 12%) were diagnosed and managed as outpatients. Demographics and baseline characteristics are shown by RDW quartile.

Median RDW was 13.6% [12.8-14.6]. Higher RDW quartile (Table 1) was associated with older age (56 vs 62 vs 63 vs 71 years, *P*_trend_<0.001), prevalence of cardiovascular disease (11% vs 16% vs 11% vs 37%, P_trend_<0.001), leukocyte count (6.1 vs 6.4 vs 6.1 vs 8.7 x10^3^/µL, *P*_trend_ =0.006), lower hemoglobin (13.8 vs 13.7 vs 12.4 vs 10.9 g/dL, *P*_trend_<0.001), and higher neutrophil count (4.3 vs 4.5 vs 4.2 vs 6.7 x10^3^/µL, *P*_trend_=0.001). Patients in the higher quartiles of RDW were more likely to be admitted to the hospital (53% vs 62% vs 57% vs 76%, P=0.014).

**Table 1.** Characteristics of Study Patients by Quartile of Red Cell Distribution Width (at Presentation)

RDW	Quartile 1 <12.9% (n=73)	Quartile 2 12.9-13.6% (n=74)	Quartile 3 13.7-14.6% (n=65)	Quartile 4 >14.6% (n=70)	*P* value (overall comparison)*	*P* value (Trend)^¥^
Age, years	56 ± 16	62 ± 17	63 ± 17	71 ± 13	<0.001	<0.001
Male	42 (58%)	37 (50%)	35 (54%)	23 (33%)	0.018	
Race					0.09	0.09
White	54 (74%)	49 (66%)	33 (51%)	37 (53%)		
Black	17 (23%)	23 (31%)	29 (45%)	30 (43%)		
Other	2 (2.7%)	2 (2.7%)	3 (4.6%)	3 (4.3%)		
Diabetes, n (%)	12 (16%)	13 (18%)	17 (26%)	17 (24%)	0.40	0.14
Hypertension, n (%)	15 (21%)	23 (31%)	15 (23%)	25 (36%)	0.16	0.11
Lung disease, n (%)	6 (8.2%)	11 (14.9%)	12 (18.5%)	8 (11.4%)	0.32	0.46
Cancer, n (%)	2 (2.7%)	2 (2.7%)	3 (4.6%)	1 (1.4%)	0.74	0.81
Cardiovascular Disease, n (%)	8 (11%)	12 (16%)	7 (11%)	26 (37%)	<0.001	<0.001
Care level, n (%)					0.014	0.18
Ambulatory	7 (9.6%)	6 (8%)	13 (20%)	7 (10%)		
Emergency department	27 (37%)	22 (30%)	15 (23%)	10 (14%)		
Admission	39 (53%)	46 (62%)	37 (57%)	53 (76%)		
Leukocyte count (x10^3^/µL)	6.1 ± 2.7	6.4 ± 3.1	6.1 ± 2.6	8.7 ± 5.6	<0.001	0.006
Lymphocyte count (x10^3^/µL)	1.15 ± 0.66	1.22 ± 0.70	1.13 ± 0.52	1.22 ± 0.87	0.84	0.86
Hemoglobin (g/dL)	13.8 ± 1.9	13.7 ± 1.6	12.4 ± 1.8	10.9 ± 2.1	<0.001	<0.001
Mean Corpuscular Volume (fL)	90.4 ± 5.4	90.6 ± 6.3	88.5 ± 6.0	90.3 ± 8.7	0.24	0.51
Monocytes (x10^3^/µL)	0.52 ± 0.25	0.49 ± 0.22	0.50 ± 0.27	0.56 ± 0.36	0.41	0.78
Neutrophils (x10^3^/µL)	4.3 ± 2.6	4.5 ± 3.0	4.2 ± 2.4	6.7 ± 5.0	<0.001	0.001
Neutrophils to Lymphocyte ratio	5.6 + 5.6	5.1 + 5.7	4.9 + 4.7	7.1 + 5.4	0.12	0.009
Platelets (x10^3^/µL)	195 ± 63	206 ± 78	193 ± 67	227 ± 116	0.07	0.15

* Overall comparison with chi-square test for categorical variables and ANOVA for continuous variables

¥ Trend test comparison with Mantel-Haenszel test for categorical variables and Spearman rank test for continuous variables

**Figure 1.**
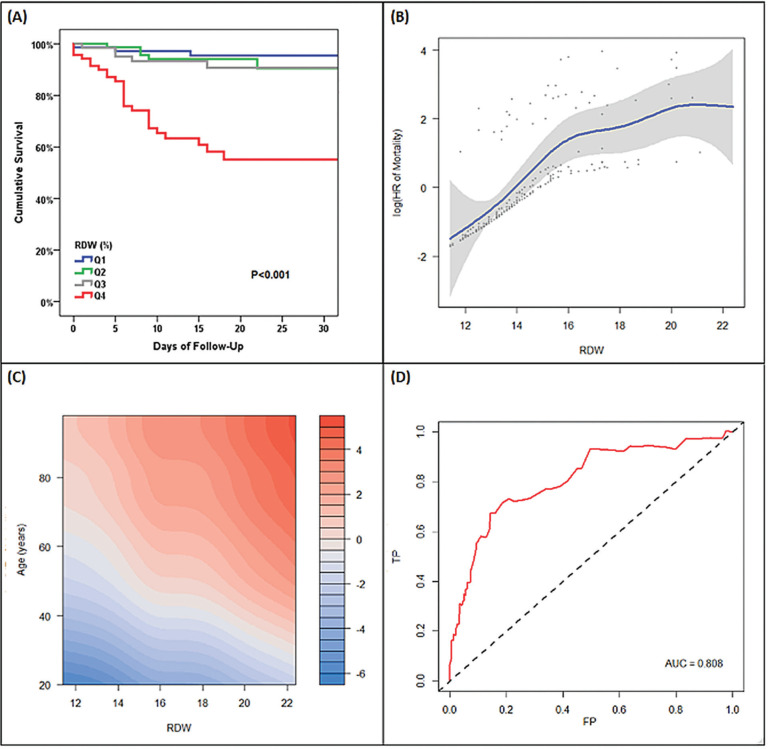
Association Between Continuous RDW and Mortality (A) unadjusted Kaplan-Meier curves, (B) unadjusted Cox regression model with RDW modelled using a penalized smoothed spline, (C) penalized-smoothed spline modelled age and RDW (color code represents log (Hazard Ratio) of mortality). Red demonstrates higher hazards of mortality and blue demonstrates lower hazards of mortality. This is modelled by age and RDW showing that 2% increase in RDW is equivalent to 10 year age increase with respect to mortality (D) Receiver Operating Characteristic curve for RDW to predict 30-day mortality (using Kaplan-Meier method) Abbreviations: HR=hazard ratio; RDW=red cell distribution width; TP = true positive rate; FP = false positive rate; AUC=area under the curve

At a median follow-up of 20 [12-25] days, 39 patients died (2 were initially managed in an ambulatory setting, 2 were initially managed in the emergency department, and 35 were hospitalized), with a cumulative mortality rate of 22%. Mortality rate was 6.9% in RDW quartile 1, 11.0% in RDW quartile 2, 13.2% in RDW quartile 3, and 53.6% in RDW quartile 4. Figure 1A shows the Kaplan-Meier survival curve by quartile of RDW. Mean survival was 38.5 (95% CI: 37-40) days in the first quartile, 33.8 (95% CI: 32-36) days in the second quartile, 37 (95% CI: 35-40) days in the third quartile, and 22 (18-25) days in the fourth quartile.

When RDW was treated as a continuous variable, there was a semi-linear association between RDW and hazard of mortality (figure 1B). The relationship between RDW and hazards of mortality by age group is shown in figure 1C. This figure suggests that there is a continued relationship between RDW and mortality in all age groups, and that a 1% increase in RDW confers a similar mortality effect to a 10-year increase in age. There was no interaction between RDW and age (*P*_interaction_=0.83), further suggesting a uniform association between RDW and mortality across the age spectrum. RDW, as a standalone marker, had good predictive power for short-term mortality with an area under the receiver operating characteristics curve (ROC AUC) of 0.81. The ROC curve is shown in figure 1D. RDW of 13.6% had 80% sensitivity and 59% specificity to predict mortality, while RDW of 14.5% (optimal cut-point) had 72% sensitivity and 81% specificity to predict 30-day mortality. As a comparator, age had a lower AUC of 0.75. Age of 63 years had 80% sensitivity and 58% specificity to predict 30-day mortality, while age of 74 years had 50% sensitivity and 80% specificity to predict 30-day mortality. Age 67 years was the optimal cut-off point, with a sensitivity of 77% and specificity of 68%.

**Figure 2.**
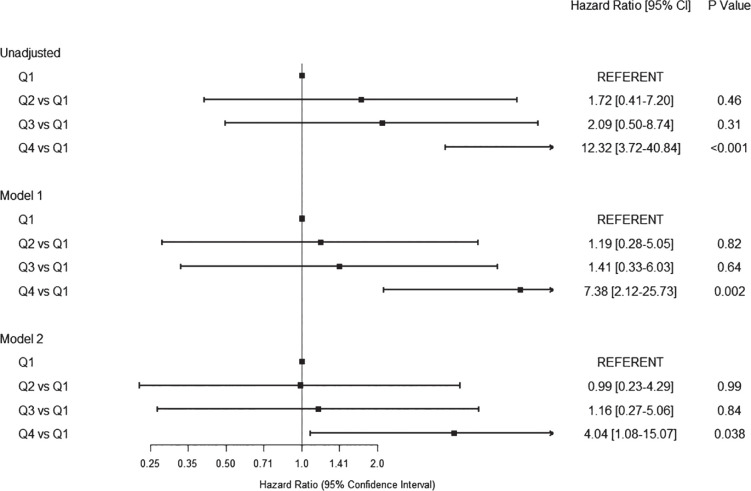
Forest Plot Depicting the Association Between Quartiles of Acute RDW and Mortality in Three Models Unadjusted; Model 1 (adjusted for age, sex, race); Model 2 (adjusted for age, sex, race, cardiovascular disease, and hemoglobin)

Compared with Q1, patients in Q4 had an increased risk of mortality (Hazard Ratio 12.32 [3.72-40.84], *P*<0.001) without significant attenuation after adjusting for age, sex, and race in model 1 (HR 7.38 [2.12-25.73], *P*=0.002), or further adjustment for hemoglobin and cardiovascular disease in model 2 (HR 4.04 [1.08-15.07], *P*=0.038). Figure 2 shows a forest plot of these hazards by quartile of RDW.

Each 1% increment in RDW was associated with a 45% relative increase in mortality (HR 1.45, [1.30-1.60], *P*<0.001), which remained unchanged after adjusting for age, sex, and race in model 1 (HR 1.49 [1.32-1.68], *P*<0.001), and further adjusting for CVD and hemoglobin levels in model 3 (HR 1.39 [1.21-1.59] per 1% increment, *P*<0.001), or after further adjustment for neutrophil count (HR 1.32 [1.11-1.58], *P*=0.002).

### Subgroup analysis of patients with remote RDW:

**Figure 3.**
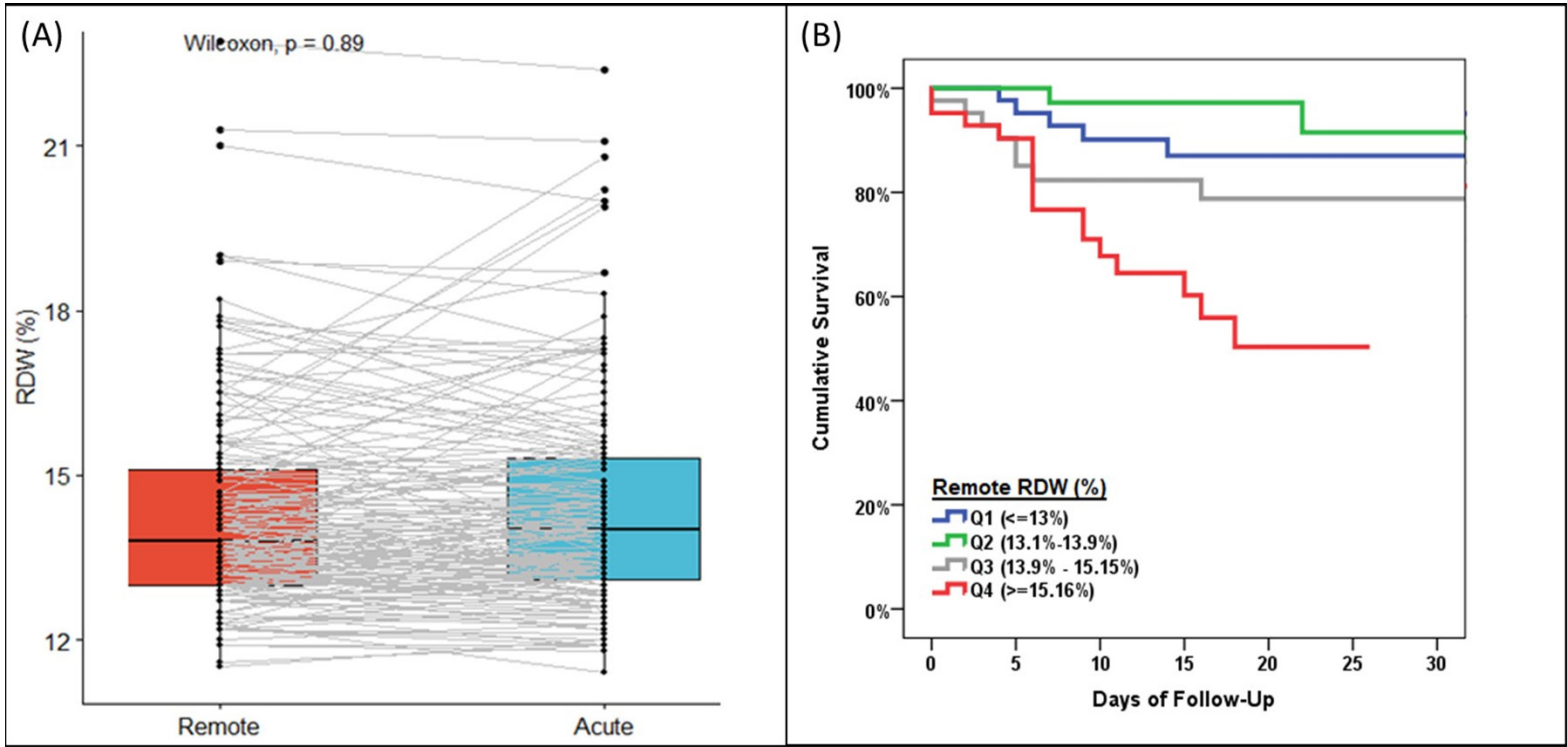
Remote RDW Is Associated With Acute RDW and Mortality (A) Change in RDW (remote to acute) in COVID-19 infected patients, (B) Kaplan-Meier test of mortality in COVID-19 infections

Of the 282 patients included in this study, 168 patients additionally had remote RDW (value measured between 7 days and 6 months prior to presentation) and acute RDW (value measured <7 days from time of COVID-19 diagnosis). There was no difference in RDW between remote values vs acute RDW: 13.6% [12.8-14.6] vs 13.9% [13.0-15.2], *P*=0.89 (figure 3A). Remote pre-COVID-19 RDW was also associated with post-COVID-19 mortality in an unadjusted model (HR 1.26 [1.11-1.44] per 1%, *P*=0.001), model 1 (HR 1.38 [1.17-1.62] per 1%, P<0.001), and model 2 (HR 1.26 [1.05-1.50] per 1%, *P*=0.011). Figure 3B shows the Kaplan-Meier survival curves by quartile of remote RDW.

**Figure 4.**
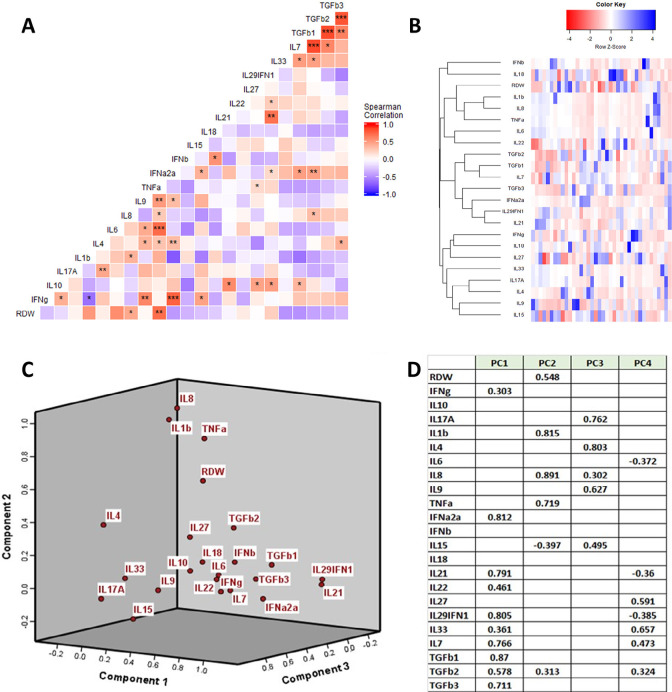
Anisocytosis in 38 Uninfected Ambulatory Adults Without Cardiovascular Disease Is Associated With a Pro-inflammatory Milieu (A) correlation matrix between RDW and cytokine profile (Spearman correlations: *P<0.05, **P<0.01), (B) hierarchical clustering showing that RDW clusters with IL1-beta, IL-6, and TNF-alpha, (C) Component plot showing that RDW clusters with TNF-alpha and IL1-beta, (D) Principal Component Analyses showing that RDW clusters with IL-1beta, TNF-alpha, and IL-8

Among the uninfected ambulatory cohort (n=38, with mean age of 56.6 ± 6.6 years, 17 females, 37 Caucasians, 21 with dyslipidemia, 9 with hypertension, 3 smokers, 0 with diabetes, 0 with malignancy, mean BMI 27.6±4.0 kg/m^2^, hemoglobin 14.5+ 1.6 g/dL, lymphocyte count 1.8 + 0.6 x10^3^/µL, neutrophils 3.9 + 1.5 x10^3^/µL, monocytes 0.5 + 0.2 x10^3^/µL). Plasma cytokines were analyzed to determine the relationship between RDW and immune activation. RDW was most closely associated with TNF-α (Spearman's rho=0.498, *P*=0.001) and IL8 (Spearman's rho=0.352, *P*=0.03) in bivariate correlations (figure 4A). Hierarchical clustering (figure 4B) and Principal Component Analyses (PC2) also demonstrated that RDW is closely linked with pro-inflammatory cytokines (IL1-beta, IL-8, and TNF-α, figure 4C-4D), but largely distinct from type 1 interferons, homeostatic, or regulatory factors (PC1).

## DISCUSSION

Our data demonstrate several novel observations. We find that those with anisocytosis (elevated RDW) are at risk for short-term mortality due to COVID-19. This relationship is independent of clinical risk factors, including cardiovascular disease. Furthermore, anisocytosis (and its associated risk) appears to predate COVID-19 because the RDW at the time of diagnosis was similar to patients' most recent pre-infection values. We also find that, in uninfected ambulatory adults without cardiovascular disease or COVID-19, an elevated RDW is associated with an altered cytokine milieu consisting of heightened pro-inflammatory cytokines, but without alterations in basal levels of type 1 interferons or homeostatic/regulatory cytokines. Thus, we conclude that, in the context of COVID-19, RDW may identify patients at heightened risk for progression to cytokine storm, and this may be due to a preexisting predilection for inflammation.

There is increasing evidence that patients with severe COVID-19 have greater inflammation. In a study of 21 patients with confirmed COVID-19 infection from Wuhan, China, severe cases (n=11) had higher levels of CRP, IL-2R, IL-6, IL-10, and TNF-α than moderate cases (n=10).

Additionally, T lymphocytes, CD4+ T cells, and CD8+ T cells decreased in nearly all patients, but were markedly lower in severe cases than in moderate cases. The expression of IFN-γ by CD4+ T cells was also lower in severe versus moderate cases [4]. However, the extent to which cytokine elevations are cause or consequence of a failing host response will require extensive mechanistic investigation, including the results of ongoing interventional trials. Our results add to the list of laboratory abnormalities that can be considered in the triage of patients, or when considering a patient's candidacy for advanced therapies. However, RDW demonstrated good discrimination and is a highly pragmatic test, widely available during routine care at little cost.

To our knowledge, no published study has identified RDW as a marker of mortality in COVID-19 infection. A prior study of patients from Wuhan, China using machine learning approaches identified RDW as a potential marker to identify severe COVID-19 infection [18]. A smaller non-peer-reviewed preprint of a study of 45 Chinese patients with COVID-19 showed that RDW and neutrophil to lymphocyte ratio can discriminate between moderate and severe disease [19].

Previous studies have reported that elevated RDW is associated with mortality in patients with critical illness and a variety of other disease states, including cardiovascular and cerebrovascular disease [9,12,20]. Our group recently showed that the risk of RDW extends to the general healthy population as well [21]. In a study of 31,178 ambulatory individuals from the National Health and Nutrition Examination Survey (1999-2010) that was followed for 12 years, RDW and ALC were independently associated with mortality, suggesting that putative CBC markers can be associated with risk of mortality. To our knowledge, this is the first report of RDW linkage with COVID-19 mortality.

Several groups, including our own, have previously demonstrated a correlation between RDW and IL-6 levels, as well as TNF-α in one report [9,12,15,17]. This may be plausible, because IL-6 can induce hepcidin expression and TNF-α has been linked to erythropoietin resistance [12,22]. Syndromes of acutely dysregulated inflammation, such as sepsis, are often accompanied by suppression of erythrocyte maturation [23]. We also studied a cohort of patients with chronic HIV infection wherein RDW retained its association with cardiovascular disease risk. In this cohort of virally-suppressed HIV-infected patients, RDW also correlated with IL-6, CD4+38+DR+T cells, and CD4+PD1+T cells [14]. A potential relationship with checkpoint molecule expression was independently corroborated in another study of 158 patients with HIV enrolled in the Hawaii Aging with HIV-Cardiovascular study, where authors demonstrated that RDW was associated with higher frequencies of CD38+HLA-DR+T cells, single TIGIT+, and dual expressing of TIGIT+PD1+, TIGIT+TIM3+, and TIM3+PD1+CD8+ T-cell subsets [24]. Thus, the relationship between RDW and immune dysregulation is undoubtedly complex, and deserves closer scrutiny in follow-up studies. However, our current study adds to this literature in that we find that relationships between RDW and pro-inflammatory factors may precede illness onset. Interestingly, in this study we show that RDW does not significantly change with acute presentation of COVID-19 infection, suggesting that it may act as a pro-inflammatory signature that increases the individual's vulnerability to COVID-19-related adverse events. As RDW is commonly reported and there is a readily available test, it has unique utility in identifying patients at high risk for progression of COVID-19 to severe disease and death. These patients can be targeted with prevention strategies, post-exposure prophylaxis, vaccinations, and early interventions to reduce mortality.

This study is limited by its retrospective study design and relatively small sample size. There may be a bias toward ordering a complete blood count in patients who are sicker, a factor which is reflected by the high percentage of patients managed in the hospital setting (ED or inpatient). We lack data on iron studies, sickle cell anemia, and other factors that may have confounded the correlation with mortality. Therefore, findings need further validation before wide adoption in clinical practice.

## CONCLUSION

In patients with COVID-19, an elevated RDW is associated with a higher risk of short-term mortality. If validated prospectively, the RDW may be a convenient, pragmatic biomarker of risk of progression to severe disease and short-term mortality in patients with COVID-19.
